# The Right to Refuse: Understanding Healthcare Providers’ Perspectives on Patient Autonomy in Emergency Care

**DOI:** 10.3390/healthcare11121756

**Published:** 2023-06-15

**Authors:** Ahmed M. Al-Wathinani, Dennis G. Barten, Hind Alsahli, Anfal Alhamid, Waad Alghamdi, Wadha Alqahtani, Raghad Alghamdi, Mohammad Aljuaid, Nawaf A. Albaqami, Krzysztof Goniewicz

**Affiliations:** 1 Department of Emergency Medical Services, Prince Sultan bin Abdulaziz College for Emergency Medical Services, King Saud University, Riyadh 11451, Saudi Arabia; naalbaqami@ksu.edu.sa; 2 Department of Emergency Medicine, VieCuri Medical Center, 5912 BL Venlo, The Netherlands; dbarten@viecuri.nl; 3 Pharmaceutical Care Division, King Faisal Specialist Hospital and Research Center, Riyadh 11211, Saudi Arabia; halsahli@kfshrc.edu.sa; 4 Primary Care Clinic and Emergency Department, Dental University Hospital-KSUMC, King Saud University, Riyadh 11451, Saudi Arabia; aalhamid2@ksu.edu.sa; 5 Department of Respiratory Services, King Abdulaziz Medical City, Ministry of National Guard, Riyadh 1154, Saudi Arabia; alalialgamdiwa@mngha.med.sa (W.A.); alghamdira16@mngha.med.sa (R.A.); 6 Department of Health Administration, College of Business Administration, King Saud University, Riyadh 11541, Saudi Arabia; 7 Department of Security Studies, Polish Air Force University, 08-521 Dęblin, Poland; k.goniewicz@law.mil.pl

**Keywords:** patient autonomy, treatment refusal, emergency care, prehospital care, healthcare providers, ethical dilemmas

## Abstract

Healthcare providers in prehospital care and emergency departments are often at the frontline of medical crises, facing a range of ethical dilemmas, particularly when it comes to patients refusing treatment. This study aimed to delve into the attitudes of these providers toward treatment refusal, unearthing the strategies they employ in navigating such challenging situations while actively working in prehospital emergency health services. Our findings showed that, as the participants’ age and experience increased, so did their inclination to respect patient autonomy and avoid persuading them to change their decision about treatment. It was noted that doctors, paramedics, and emergency medical technicians demonstrated a deeper understanding of patients’ rights than other medical specialists. However, even with this understanding, the prioritization of patients’ rights tended to diminish in life-threatening situations, giving rise to ethical dilemmas. This underlines the complexity of balancing the healthcare professionals’ responsibilities and the patients’ autonomy, which can generate ethically challenging scenarios for those working in emergency healthcare. By investigating these attitudes and experiences, this study seeks to foster a more profound understanding of the ethical quandaries faced by emergency healthcare providers. Our ultimate aim is to contribute to the development of effective strategies that support both patients and professionals in managing these tough circumstances.

## 1. Introduction

Emergency medical services, which provide prehospital care—a critical type of medical attention given before a patient reaches the hospital—alongside emergency departments are integral components of healthcare systems worldwide. Both these components play a crucial role in ensuring the health and wellbeing of citizens [1]. Many countries, including Saudi Arabia, provide free access to various levels of public healthcare services for their citizens. As an example, Saudi Arabia’s government-funded healthcare system allocated a budget of USD 568 million to prehospital care in 2016 [2], and by 2019, the country operated 2386 ambulances and service vehicles that addressed 569,209 cases, with approximately two-thirds of those cases being transferred to emergency departments [3]. Trauma-related emergency department visits, including burns and injuries to the head, chest, or spine, accounted for 101,274 cases in Saudi Arabia that year [3].

The unique characteristics of emergency care, such as its time-sensitive nature and the urgency of decision-making, distinguish it from hospital-based care [4]. Delays in treatment can exacerbate a patient’s condition, leading to adverse outcomes. Despite the critical role of decision-making in this setting, research examining the ethical dilemmas faced by healthcare professionals in emergency care is limited globally [5].

Treatment refusal poses a recurrent challenge to healthcare professionals in many countries. For example, from September to October 2022, treatment refusal was noted in 8% to 10% of cases in Saudi Arabia, with as many as 19% of doctors reportedly encountering treatment refusal in their daily practice [5,6]. Furthermore, in complex situations, ambulance nurses often chose to treat patients as they would want to be treated themselves, indicating the inherent ethical dilemmas [7].

A study conducted in Spain found that, while prehospital emergency health professionals generally respected patients’ right to refuse treatment, this respect was not prioritized in life-threatening situations or when patients lacked decision-making capacity. In these situations, professionals adopted a more paternalistic approach [8]. This balance between respecting a patient’s right to refuse treatment and the responsibility to provide medical care is a core ethical challenge faced by healthcare providers across the globe.

In the context of Saudi health law, as in many other countries, treatment refusal is recognized as one of the patients’ rights. Refusal of treatment is acknowledged as a fundamental patient right within the purview of Saudi health law [9,10]. Patients are even allowed to choose their physicians based on available resources at the healthcare facility [11]. This invariably places healthcare providers in a quandary between respecting patient autonomy and fulfilling their professional responsibility to provide treatment. This study seeks to examine this complex issue in the broader context, focusing on refusal of diagnosis, follow-up, protection, and hospital transfer, in addition to treatment refusal, by exploring the attitudes of prehospital care and emergency department health professionals in Saudi Arabia toward patients’ refusal of treatment. The goal is to understand the attitudes of prehospital and emergency health professionals in emergency departments worldwide toward patients’ refusal of treatment using Saudi Arabia as a case study.

## 2. Materials and Methods

### 2.1. Study Location

Although citizens in Saudi Arabia have free access to all levels of public healthcare services, funded by the central government, the healthcare system in Saudi Arabia is diverse and multifaceted. Our study, conducted from September to October 2022, reflects this diversity by including private hospitals, Ministry of Health (MOH) government hospitals, and non-MOH government hospitals in Riyadh, the capital city of Saudi Arabia.

Private hospitals in Saudi Arabia are privately owned and operate on a fee-for-service basis. They provide an alternative for individuals who opt for private health insurance or can afford to pay out-of-pocket for their care. Despite their private status, these hospitals offer a range of services that often mirror those available in public hospitals, including emergency care.

Government hospitals, managed by the Ministry of Health (MOH), constitute the backbone of public healthcare in the country, providing comprehensive care to the majority of the population. In addition to MOH-managed facilities, non-MOH government hospitals exist, which are public hospitals managed by other government bodies, such as military or security forces, universities, or the royal court. These hospitals cater to specific segments of the population and their dependents, such as military personnel or university students.

Riyadh, with a population of 6.5 million, represents approximately one-sixth of Saudi Arabia’s total population and encompasses an area of 3115 square kilometers. This region is home to a significant healthcare infrastructure with 49 healthcare facilities, including hospitals and clinics. These facilities collectively employ a total of 7593 emergency department staff, as reported by the General Authority for Statistics of KSA [12]. This comprehensive inclusion of varied healthcare settings in our study not only captures the diversity of the Saudi Arabian healthcare system but also enhances the generalizability of our research findings.

### 2.2. Study Participants

We followed the total coverage sampling technique of all emergency healthcare workers who fulfilled the inclusion criteria and accepted to participate in this study. The inclusion criteria were emergency healthcare workers caring for patients in prehospital and emergency health services with professional affiliations. Stratified sampling was performed to achieve variation in age, gender, and professions. Due to the disproportionality of employment in ED (predominance of professions other than physician), these results may seem misleading. However, the employment system in emergency departments differs due to the amount of work and responsibilities, which is why it is less often chosen by physicians.

Not being employed in prehospital and emergency health services was considered as a criterion for exclusion. The sample size was determined using the following formula: n = z^2^pq\e^2^ (n = sample size, z = z score for 95% confidence interval, p = estimated proportion, q = p − 1, e = margin of error). The confidence level was determined to be 95%, an estimated proportion of 50% was specified, a 6% margin of error was set, and an estimated population of 7593 was used (the estimated population is number of emergency department staff working in Riyadh). The minimum required sample size was calculated to be 258.

Considering responsiveness and cooperation, we determined 326 individuals as the sample size (Table 1). Due to the unavailability of a few of the participants in the study area during the study period for several reasons, including refusal to participate in this study, absence, and inability to access them online, the final sample size was 320 out of 326. The response rate was 98.15%.

Written informed consent was obtained from the participants before collecting the data. The information included this study’s purpose, the voluntary nature of their participation, and strict confidentiality and secure data storage. The survey was anonymous, and all respondents agreed to participate in the survey. 

### 2.3. Questionnaire

The first phase of this study involved the authors engaging in a comprehensive literature review to identify the key dimensions essential for developing a robust questionnaire. This was achieved by utilizing the following keywords either individually or in combination: emergency; prehospital; treatment refusal; autonomy; ethics; decision-making; and paternalism. The data collected from PubMed, Scopus and Web of Science were meticulously organized, categorized, and mapped. To refine the scope of this review, the search was restricted to English publications. A qualitative thematic analysis, based on an inductive approach, was then applied to the selected literature. The aim of this content analysis was to identify similarities and differences in the findings across the articles, which would ultimately serve as the foundation for the questionnaire design.

The resulting questionnaire was divided into two sections. The first section comprised five questions that sought information on the participants’ personal and professional characteristics, including age, gender, role, experience, and nationality. The second section contained ten statements related to issues surrounding treatment refusal. Each statement in this section was designed to be answered using a Likert scale, ranging from 1 (strongly disagree) to 5 (strongly agree). A detailed overview of the items is provided in Table 2.

For the second phase of this study, a pilot test was conducted with a group of 10 medical employees from King Saud University hospital to evaluate the questionnaire. To avoid potential bias, these individuals were excluded from the main study, and their responses were not included in the final data analysis. The questionnaire was then refined based on a comprehensive review of its logic, relevance, comprehension, legibility, clarity, and usability. The estimated time to complete the questionnaire ranged from 5 to 10 min.

The questionnaire was disseminated to the participants via direct email, with a unique link to the survey tool.

### 2.4. Data Collection

Due to the ongoing COVID-19 pandemic and internal health safety regulations, data were collected via an online questionnaire distributed to emergency departments in all hospitals in Riyadh, KSA. A total of 326 participants were provided with the survey, with 320 completing it.

### 2.5. Statistical Analysis

Participants’ personal information was analyzed using descriptive analysis with Excel. This software was chosen due to its widespread use, accessibility, and user-friendly interface, which enabled the efficient organization and analysis of basic demographic data. Contingency tables and chi-square tests were employed to compare age, gender, experience, and duty across the ten statements. These analyses were performed using Jamovi (version 1.2) 2020 and R comprehensive computer software (version 3.6) 2019.

The selection of Jamovi and R software for the statistical analysis was driven by several factors. Jamovi is a free, open-source statistical software that integrates with R, offering an intuitive and user-friendly interface for conducting a wide range of advanced statistical analyses [13], and R is a popular, open-source programming language and software environment for statistical computing and graphics, which has been widely adopted in various fields of research [14]. Both software packages have been employed in studies with similar methodological approaches [15,16]. Their extensive documentation, active user communities, and the availability of numerous packages for specialized analyses make them well-suited for this study.

### 2.6. Ethical Considerations

This study was approved by the King Saud University Research Center Institutional Review Board (Rf No.: KSU-HE-22-639). Agreement for participation was obtained from each participant before answering the questionnaire.

## 3. Results

The total number of participants in this study was 326. However, six participants were excluded due to incomplete data or refusal to participate. The demographic data of the participants are presented in Table 1. Among the 320 participants, 96.3% were Saudi, and 3.70% were non-Saudi. The majority of participants (57.5%) were between 20 and 29 years old. Males represented 55.9% of participants. The largest professional group in this study was paramedics (24.7%), followed by respiratory therapists (24.4%) and nurses (23%). In terms of experience, the largest proportion of participants had 3 to 5 years of professional experience (25.3%), followed by those with 5 to 10 years of experience (20.6%).

Table 2 displays the mean, standard deviation, mode, and score for each statement. A comprehensive analysis of the statements reveals that the second and fifth statements received the highest scores, with a mean of 5, indicating strong agreement. Additionally, the first, fourth, eighth, and tenth statements scored 4, with modes ranging from 4 to 5, suggesting agreement to strong agreement. Most participants responded neutrally to the third and ninth statements. Lastly, both the sixth and seventh statements had a score of 2, with modes of 2 (disagree) and 1 (strongly disagree). The statements were categorized into four groups: the first two statements concerned patients’ rights; the third, fourth, and seventh statements addressed refusal of treatment in emergency situations; the fifth, sixth, and tenth statements pertained to healthcare providers’ attitudes regarding treatment refusal; and the eighth and ninth statements dealt with paternalism.

Table 3 presents the number and percentages of the five agreement levels for these categories.

The analysis based on age revealed a significant correlation with the sixth statement, which asserts that healthcare providers should not try to persuade patients who refuse treatment to change their decision (*p*-value 0.008). Although more participants disagreed with this statement, the level of agreement increased with age. For instance, among those aged 20–29, 7.6% strongly agreed while 32.6% strongly disagreed; in contrast, 9.3% strongly agreed and 12.7% strongly disagreed among those aged 30-39.

When comparing responses based on experience, associations were found between statements 3, 6, and 8 and years of experience. However, only the response to the sixth statement showed statistical significance, with agreement levels rising as experience increased (*p*-value 0.001).

Comparisons based on gender did not yield any significant correlation. However, a higher percentage of males strongly agreed with paternalistic statements compared to females.

Comparing scores according to healthcare professionals’ duties revealed that the response to the second statement differed significantly when comparing doctors, paramedics, and EMTs with other health providers (*p*-value 0.001).

## 4. Discussion

Encountering patients who refuse ambulance services can precipitate ethical dilemmas for healthcare professionals [17]. Balancing respect for patient autonomy—the right of patients to make their own medical care decisions, including refusal of treatment or hospital transfer—with the necessity to provide appropriate medical care can prove challenging [17,18].

The reasons behind refusal of medical attention or discharge against medical advice are manifold and complex, ranging from misunderstandings and language barriers to denial of illness, mistrust of the healthcare system, previous unpleasant experiences, fear, anger, and family obligations [19,20,21,22,23,24,25,26,27,28]. It is vital for healthcare providers to understand these factors to better address them and provide optimal patient care.

Within the framework of clinical practice, two types of consent are recognized: implied and informed consent. While implied consent covers life-saving procedures in urgent situations, informed consent safeguards patient autonomy and their right to make decisions affecting their bodies [29,30]. In prehospital and emergency care, conflicts can arise, with healthcare providers often unsure whether a patient fully comprehends the implications of refusing medical care and when the patient loses their decision-making capacity.

When faced with a patient who refuses treatment, healthcare providers have limited options that are largely contingent on the patient’s mental competency. They can respect the patient’s decision, attempt to persuade the patient otherwise, or provide treatment despite the patient’s refusal. However, the latter two options either require exceptional communication skills or disregard patient autonomy [31].

In the process of assessing treatment refusal, it is essential to consider the patient’s capacity to make informed decisions. Medical situations can be complex, particularly in acute settings in which conditions such as delirium or acute mental health issues can affect a patient’s capacity [32]. According to Huber et al., a well-defined algorithm is needed when using the Guardianship Act, the Mental Health Act, and the Public Health Act in emergency departments [32]. Their work highlights the need for tools and guidelines to help healthcare professionals navigate these difficult situations and respect the patient’s autonomy while ensuring their safety. In this context, our study’s findings suggest that further research is needed to develop strategies that balance these critical elements in the Saudi Arabian emergency healthcare setting [32].

Our study findings demonstrate that over 90% of participants agreed with patient autonomy toward treatment refusal. Furthermore, 83.4% strongly agreed that providers must furnish the patient with clear and detailed information regarding the possible consequences of their decision. However, attitudes diverged in life-threatening situations, with a significant percentage of participants not supporting a patient’s right to refuse treatment [33,34,35,36].

Experiences and professional roles can impact decision-making mechanisms and attitudes toward treatment refusal. For instance, it has been found that ambulance personnel with more years of experience demonstrated significant differences in decision-making mechanisms [34]. Similarly, staff in different professional roles such as doctors, paramedics, and EMTs were found to have varying awareness levels about refusal of treatment as a fundamental patient right [34,35,36,37].

The ongoing COVID-19 pandemic has substantially affected the practice of limiting therapeutic intervention, underscoring the imperative for frameworks such as “Transitional Justice”. Transitional justice, as defined by Gready and Robins [38], refers to mechanisms aimed at redressing massive human rights abuses and establishing societal reconciliation. In the context of healthcare, it helps uphold fundamental ethical principles such as patient autonomy and non-maleficence. In parallel, dialogues concerning do-not-resuscitate (DNR) orders and one-sided DNR decisions have been thrust into the spotlight, further necessitating the need to delve into these complex ethical challenges.

Treatment refusal can potentially exacerbate a patient’s condition, and studies have shown adverse outcomes in such cases [39,40]. Therefore, it is essential to establish clear protocols on managing patient refusal. These protocols should account for the patient’s mental capacity, provide comprehensive information, and respect the patient’s autonomous choices [41,42].

In the context of the Kingdom of Saudi Arabia, refusal of treatment is not an isolated phenomenon but reflects a global challenge that healthcare providers encounter [43]. Therefore, our findings can offer valuable insights for healthcare systems worldwide, contributing to a more comprehensive understanding of ethical dilemmas in healthcare and shaping policies and protocols to improve patient care.

Understanding and addressing the ethical dilemmas associated with treatment refusal requires healthcare providers to engage in continuous education and communication with patients [44]. Institutions should offer training programs focusing on the ethical aspects of healthcare, including patient autonomy, informed consent, and decision-making capacity [45,46].

In an era in which the medical landscape is constantly evolving due to factors such as the COVID-19 pandemic, healthcare professionals must remain vigilant and adaptable in navigating ethical challenges [47]. The pandemic has brought about significant changes in the way healthcare is delivered, with an increasing focus on telemedicine and remote consultations [48,49]. This shift necessitates a reevaluation of the traditional ways of addressing treatment refusal and ensuring that patient autonomy and non-maleficence are maintained.

Moreover, interdisciplinary collaboration among healthcare professionals is essential in developing comprehensive guidelines and policies that can help manage complex ethical situations. Input from various stakeholders, such as ethicists, lawyers, and patient advocacy groups, can enrich the understanding of ethical issues and ensure that the policies developed are in the best interest of the patients [50].

Public awareness campaigns focusing on patient rights and responsibilities can also play a crucial role in minimizing ethical dilemmas related to treatment refusal [29]. By fostering an environment in which patients are well-informed about their rights and the potential consequences of refusing treatment, healthcare providers can better support patient autonomy while ensuring that patients receive the necessary care.

## 5. Limitations

This study carries inherent limitations, an important one being the concentration of selected hospitals solely in Riyadh. As Riyadh is the capital of Saudi Arabia and holds a higher density of healthcare facilities compared to other regions in the country, the findings may not precisely reflect the experiences of healthcare professionals and patients in different regions where access to healthcare facilities and resources varies.

The sample size of this study is another limiting factor. The small size might not comprehensively represent the experiences and perspectives of all healthcare professionals in the emergency departments across Saudi Arabia. Moreover, the scarcity of local literature on treatment refusal in a prehospital setting limits the broader applicability of our findings.

Further, the cross-sectional design of this study limits its scope as it captures data at a single point in time, making it incapable of establishing causality. It would be beneficial to carry out future research utilizing a longitudinal design to track changes in treatment-refusal rates and the influencing factors over time.

In addition, while we believe our findings have international relevance, we must acknowledge that without similar studies conducted in other global contexts, this belief remains speculative. Therefore, international research in this area would be advantageous to confirm or challenge the universal applicability of our results.

Despite these limitations, this study provides valuable insights into the treatment-refusal issue among the Saudi population. It underscores the necessity for more extensive research to comprehend this complex issue better and devise effective strategies to address it in the healthcare sector.

## 6. Conclusions

Our study’s findings underscore the paramount importance of acknowledging and upholding patients’ rights, including providing detailed information about their treatment options, particularly within the challenging environment of emergency departments. In the context of Saudi Arabia, healthcare practitioners demonstrated strong support for the patient’s right to refuse treatment, even though this stance could become complicated in critical, life-threatening scenarios. The results suggest a need for a nuanced approach to medical decision-making, one that respects autonomy while also considering the potential for dire health outcomes. Herein lies an ethical conundrum that warrants further exploration and understanding. The complexities of balancing patient autonomy, non-maleficence, and beneficence in emergency settings cannot be overstated, especially when refusal of care may lead to substantial health risks. The critical implications of this study highlight the need for more comprehensive strategies to enhance patient–healthcare provider communication, particularly in emergency settings. The goal is not merely to inform patients about their conditions and potential interventions, but to foster an environment that promotes shared decision-making. This concept entails a two-way exchange, wherein patients are empowered to voice their values and preferences, and healthcare providers employ their expertise to contextualize medical information and options. Our findings also underscore the relevance of continual professional development for healthcare providers, focusing on ethical considerations in healthcare delivery. This is particularly important in the context of a rapidly evolving healthcare landscape, as seen during the COVID-19 pandemic, which has prompted adaptations in healthcare delivery, and in turn, necessitated a reevaluation of how we approach ethical dilemmas, such as treatment refusal. Furthermore, this study indicates the necessity of implementing clear protocols to navigate complex ethical situations, such as treatment refusal, effectively. These protocols ought to be constructed through interdisciplinary cooperation and input, considering the diverse perspectives of all stakeholders. In the final analysis, it is vital to amplify public understanding about patient rights and responsibilities, which can lead to more informed decision-making, particularly in circumstances in which treatment refusal is under consideration. A thoroughly informed patient can actively participate in their own care, making choices in line with their personal values and preferences, thereby fostering an ethical, patient-centered approach to healthcare delivery. The challenges tied to treatment refusal are not solely confined to Saudi Arabia but are issues of global relevance within the healthcare sector. As we push for ethical, patient-centered care, it is imperative to prioritize patient autonomy, enhance communication, encourage interdisciplinary collaboration, and guarantee that clear protocols are in place to effectively address these intricate ethical situations. Future research should take these findings as a stepping stone to endorse optimal patient outcomes and uphold the ethical principles that form the foundation of healthcare.

## Figures and Tables

**Table 1:** Demographic characteristics of participants.

Features		Number	Percent
Age	Under 20 years	2	0.6%
	20–29 years	184	57.5%
	30–39 years	118	36.9%
	40 years and over	16	5%
Total		320	100%
Gender	Female	141	44.1%
	Male	179	55.9%
Total		320	100%
Duty	EMT	6	1.9%
	Nurse	74	23.1%
	Pharmacist	22	6.9%
	Paramedic	79	24.7%
	Respiratory therapist	78	24.4%
	Health officer	6	1.9%
	Doctor (physician)	25	7.8%
	Paramedic student	11	3.4%
	Other	19	6%
Total		320	100%
Experience	Less than 1 year	63	19.7%
	1–2 years	57	17.8%
	3–5 years	81	25.3%
	5–10 years	66	20.6%
	More than 10 years	53	16.6%
Total		320	100%
Nationality	Saudi	308	96.3%
	Non-Saudi	12	3.7%
Total		320	100%

**Table 2:** The participants’ responses mean, mode, SD given for the statements regarding refusal of treatment.

Statements	Mean	SD	Mode	Score *
Q1. Patients should have the right to refuse treatment or any other medical practices provided for them.	4.25	0.79	5	4
Q2. Refusal of treatment should be assured as a fundamental patient right through legal regulations.	4.55	0.6	5	5
Q3. On principle, patients have the right to refuse treatment even in critical, life-threatening situations.	2.6	1.3	3	3
Q4. Calling an ambulance does not mean that patients accept all the medical practices without questioning or renounce their right to refuse treatment.	3.8	1.07	4	4
Q5. Health professionals should provide patients who refuse treatment with clear, comprehensive, and detailed information regarding the possible consequences of their decision.	4.7	0.53	5	5
Q6. Health professionals should not try to persuade patients who refuse treatment to change their decision.	2.3	1.19	2	2
Q7. Patients who refuse treatment should not be provided medical intervention even in a life-threatening emergency situation.	2.0	1.21	1	2
Q8. Patients who do not have enough knowledge about their situation or whose consciousness level is not sufficient to make reasonable decisions cannot use their right to refuse treatment.	3.8	1.14	4	4
Q9. Relatives of patients who lack decisional capacity or who are unconscious should have the right to refuse treatment on behalf of patients.	3.3	1.31	4	3
Q10. That a patient refuses the recommended treatment does not mean health professionals should stop monitoring them.	4.2	1.13	5	4

* 5 = strongly agree, 4 = agree, 3 = neutral, 2 = disagree, 1 = strongly disagree.

**Table 3:** The participants’ responses for statements regarding refusal of treatment categories and percentage.

The Participants’ Responses Given for the Statements	Strongly Agree	Agree	**Neutral**	**Disagree**	**Strongly Disagree**
Q1. Patients should have the right to refuse treatment or any other medical practices provided for them.	137 (42.8%)	136 (42.5%)	40(12.5)	4 (1.2%)	3 (0.9%)
Q2. Refusal of treatment should be assured as a fundamental patient right through legal regulation.	182 (56.9%)	121 (37.8%)	16 (5%)	1 (0.3%)	0
Q3. On principle, patients have the right to refuse treatment even in critical, life-threatening situations.	40 (12.5%)	37 (11.6%)	88 (27.5%)	76 (23.8%)	79 (24.7%)
Q4. Calling an ambulance does not mean that patients accept the medical practices without questioning or renounce their right to refuse treatment.	102 (31.9%)	120 (37.5%)	61 (19.1%)	24 (7.5%)	13 (4.1%)
Q5. Health professionals should provide patients who refuse treatment with clear, comprehensive, and detailed information regarding the possible consequences of their decision.	267 (83.4%)	46 (14.4%)	4 (1.2%)	1 (0.3%)	2 (0.6%)
Q6. Health professionals should not try to persuade patients who refuse treatment to change their decision.	27 (8.4%)	30 (9.4%)	62 (19.4%)	121 (37.8%)	80 (25%)
Q7. Patients who refuse treatment should not be provided medical intervention even in a life-threatening emergency.	25 (7.8%)	17 (5.3%)	39 (12.2%)	98 (30.6%)	141 (44.1%)
Q8. Patients who do not have enough knowledge about their situation or whose consciousness level is not enough to make reasonable decisions cannot use their right to refuse treatment.	110 (34.4%)	120 (37.5%)	37 (11.6%)	40 (12.5%)	13 (4.1%)
Q9. Relatives of patients who lack decisional capacity or who are unconscious should have the right to refuse treatment on behalf of patients.	65 (20.3%)	106 (33.1%)	62 (19.4%)	42 (13.1%)	45 (14.1%)
Q10. That a patient refuses the recommended treatment does not mean health professionals should stop monitoring them.	178 (55.6%)	93 (29.1%)	10 (3.1%)	22 (6.9%)	17 (5.3%)

## Data Availability

The datasets used and/or analyzed during the current study are available from the corresponding author on reasonable request.
